# Relative Skeletal Muscle Mass Is an Important Factor in Non-Alcoholic Fatty Liver Disease in Non-Obese Children and Adolescents

**DOI:** 10.3390/jcm9103355

**Published:** 2020-10-19

**Authors:** Yoowon Kwon, Su Jin Jeong

**Affiliations:** Departments of Pediatrics, CHA Bundang Medical Center, CHA University School of Medicine, Seongnam 13496, Korea; youyisi68@gmail.com

**Keywords:** non-alcoholic fatty liver disease, skeletal muscle mass, sarcopenia, muscle-to-body fat ratio, children

## Abstract

Recently, sarcopenia was identified as a risk factor for non-alcoholic fatty liver disease (NAFLD) in adults. We here investigated the association between skeletal muscle mass (SMM) and NAFLD in non-obese children and adolescents. A retrospective medical chart review was performed for individuals aged 9–15 years diagnosed with NAFLD. Healthy volunteers aged 9–15 years were recruited as controls. Participants were subject to laboratory tests, abdominal sonography, and multi-frequency bioelectrical impedance analysis. SMM data were calculated as the skeletal muscle-to-body fat ratio (MFR), and the diagnosis of fatty liver was established by abdominal sonography. The control and NAFLD groups included 73 and 53 individuals, respectively. No significant difference was observed in gender and body mass index (BMI) distribution between the groups. Mean MFR was significantly lower in individuals with NAFLD than in those without (0.83 vs. 1.04, *p* = 0.005). After adjusting for age, sex, BMI, and serum glucose, the risk of having NAFLD was significantly associated with a decreased MFR (*p* = 0.016). NAFLD is significantly associated with relatively low SMM in non-obese children and adolescents. Increasing SMM, such as weight training, can be suggested as one of the treatment strategies in pediatric NAFLD without obesity.

## 1. Introduction

Non-alcoholic fatty liver disease (NAFLD) is defined as steatosis with ≥5% fat infiltration in the liver in the absence of alcohol-, drug-, virus-associated, or other known liver pathology [1,2]. The spectrum of NAFLD ranges from steatosis to steatohepatitis (non-alcoholic steatohepatitis, NASH), fibrosis, and cirrhosis [1,3,4]. Even steatosis, minor fat infiltration, can eventually lead to progressive liver disease, a major cause of liver-related mortality, such as liver cirrhosis or hepatocellular carcinoma [5]. NAFLD is now the most common type of chronic liver disease in children and adolescents. Changes in lifestyle and environment, an unhealthy diet, and lack of physical activity have resulted in higher rates of childhood obesity; as a result, NAFLD, which is a hepatic complication of obesity and metabolic syndrome, is becoming a major public health issue in children [6]. Pediatric NAFLD is of particular concern because children have a long life expectancy and because it increases the risk of type-2 diabetes or cardiovascular disease in adulthood as well as liver-related morbidity [7].

The most important well-known risk factors for NAFLD in children include central obesity and insulin resistance [5,6]. However, not all children with NAFLD are obese [4]. According to several studies, NAFLD may occur in up to 2.3–5.0% of children with a normal body mass index (BMI) [1,8]. Therefore, it is necessary to identify risk factors associated with the development of NAFLD in non-obesity populations. For example, the role of genetic factors has been studied over the past decade. New single nucleotide polymorphisms, such as those in *PNPLA3*, *TM6SF2*, *MBOAT7*, and *GCKR*, have been identified as predictors of NAFLD in both adults and children [4,6].

Recently, sarcopenia has emerged as a new risk factor for NAFLD, and several studies on adults have revealed an association between low skeletal muscle mass (SMM) and NAFLD. SMM secretes myokines and plays a crucial role in systemic glucose metabolism, being the most abundant insulin-sensitive tissue [9]. Sarcopenia is a condition involving decreased SMM and strength. It is known to promote insulin resistance, leading to the development of metabolic syndrome and diabetes [10,11]. Although the exact mechanism between the sarcopenia and NAFLD has yet to be fully identified, several studies showed that sarcopenia was significantly associated with an increased risk of having NAFLD, and even the severity of NAFLD based on abdominal sonography grading appears to be associated with sarcopenia, regardless of visceral fatness and other metabolic confounders [10,11,12,13,14,15]. Another seven-year longitudinal study showed that increases in relative SMM over time might lead to benefits, either by preventing the development of NAFLD or by the resolution of existing NAFLD [16].

To the best of our knowledge, previous studies on sarcopenia and NAFLD have been limited to adult study subjects. If low SMM also affects the presence or severity of NAFLD in children, early intervention by strength training could be very helpful in terms of preventing a poor prognosis in NAFLD. Therefore, we aimed to investigate the link between SMM and NAFLD in children and adolescents.

## 2. Materials and Methods

### 2.1. Subjects

We reviewed the medical records of children and adolescents aged 9–15 years referred to the Bundang CHA pediatric gastrointestinal clinic between February 2014 and February 2020 due to abnormal liver function tests. Children who had received a final diagnosis of NAFLD upon abdominal sonography were selected as subjects, all of whom had data of laboratory tests, abdominal sonography, and multi-frequency bioelectrical impedance analysis (BIA; InBody720, Biospace, Seoul, Korea). Initially, a total of 178 subjects were enrolled. Among these, we excluded subjects whose BMI was ≥23 (*n* = 125).

For the control group, healthy volunteers aged 9–15 years whose BMI was <23 were recruited from two elementary schools in Seongnam-si, Gyeonggi-do. After laboratory tests and abdominal sonography, 73 children with normal liver function tests and sonography were enrolled in the study.

None of the participants had a history of alcohol consumption or positive serological markers for hepatitis B or C virus. Subjects with hepatitis B virus-positive parents or a family history of hepatitis B infection, known autoimmune hepatitis, immunocompromised status, or malignancy were excluded. Eventually, data on 53 individuals in the NAFLD group and 73 subjects in the control group were eligible for analysis.

The study was approved by the CHA University Institutional Review Board (IRB) (No.2020-08-028). Written informed consent documents were obtained from all parents or guardians of the participating children.

### 2.2. Anthropometric Measurements

For the evaluation of body composition, study participants were tested by BIA while wearing light indoor clothes and no shoes. The subjects stood on the machine for about 2 min, with their legs apart and arms slightly separated from their trunk. Various parameters were automatically measured, including height (cm), weight (kg), BMI (kg/m^2^), body fat mass (kg), segmental lean muscle mass (kg), and percentage of body fat and lean muscle mass (%).

### 2.3. Definition of Skeletal Muscle Mass (SMM) Values and Sarcopenia

Appendicular skeletal muscle mass (ASM, kg) was calculated as the sum of the SMM of the four limbs, assuming that all non-fat and non-bone tissue is skeletal muscle [9]. The BIA technique has been used to estimate ASM in recent studies since it correlates well with dual-energy x-ray absorptiometry (DEXA) and has been validated for the evaluation of body composition [16].

We calculated skeletal muscle-to-body fat ratio (MFR), ratio of SMM, and measures of fatness, as ASM/body fat mass. According to the study by Kim et al., which used the mean and standard deviation (SD) of MFR for the 3rd BMI quintile of Korean children and adolescents (i.e., cutoff value = mean value—1 SD of MFR for the 3rd BMI quintile), the MFR cutoff value for sarcopenia was defined as 1.155 for boys and 0.723 for girls [9].

### 2.4. Laboratory Measurements

Venous samples were obtained after eight hours of overnight fasting. Data on serum alanine aminotransferase (ALT), aspartate aminotransferase (AST), gamma-glutamyltransferase (GGT), fasting glucose, total cholesterol, triglyceride, high-density lipoprotein cholesterol, and low-density lipoprotein cholesterol were collected. Hepatitis B surface antigen and antibodies to hepatitis C virus were also measured. Blood samples were collected in separator tubes containing silica and a gel clot (Becton, Dickinson and Company, Franklin Lakes, NJ, USA), centrifuged, and analyzed within two hours. All laboratory tests were performed using standard laboratory methods.

### 2.5. Evaluation of NAFLD

Abdominal ultrasonography was performed by board-certified pediatric radiologists to diagnose fatty liver blinded to the clinical and laboratory results of the subjects. The sonographic diagnosis of fatty liver relied on diffusely increased liver parenchymal echogenicity (the so-called “bright liver”) compared with the adjacent kidney and spleen without focal lesion, increased attenuation of the ultrasound beam, or decreased sonographic visualization of the portal and hepatic veins [17,18]. Fatty liver severity was divided into four grades as follows: grade-0, normal; grade-1, mild NAFLD; grade-2, moderate NAFLD; grade-3, severe NAFLD, as per the published criteria of Saadeh et al. [10,19].

### 2.6. Statistical Analysis

The data were analyzed with descriptive statistics and presented as means, standard deviations, and proportions. Comparisons of mean values of continuous variables between groups were conducted by t-test; for categorical variables, chi-square tests were used. Logistic regression analysis was used to calculate beta coefficients (B), standard errors (SE), and odds ratios (OR), after adjustment for confounding factors such as age, gender, BMI, and fasting glucose data. Probability (*p*) values ≤ 0.05 were considered statistically significant. All statistical analyses were performed using IBM SPSS Statistics 25.0 (IBM^®^ SPSS^®^ Statistics Server, Armonk, NY, USA).

## 3. Results

### 3.1. Comparison of Baseline Characteristics Between The Control and NAFLD Group

The number of subjects included in the control and NAFLD groups was 73 and 53, respectively. Table 1 summarizes baseline demographic data and data on the clinical characteristics of the two groups. The mean age of both groups was 10.8 years. There was no significant difference between the two groups in the distribution of sex and BMI.

**Figure 1:**
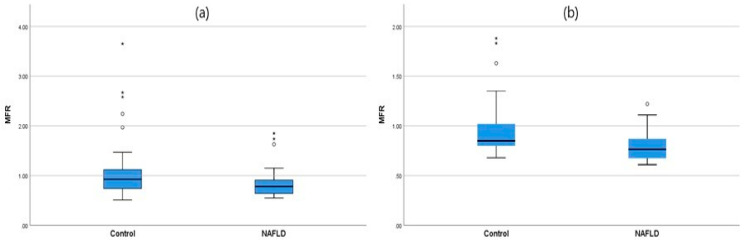
Box and whisker plot of median MFR (skeletal muscle-to-body fat ratio) in the control and NAFLD (non-alcoholic fatty liver disease) group in boys (**a**) and girls (**b**).

The mean MFR value was significantly lower in subjects with NAFLD than in those without (0.83 vs. 1.04, *p* = 0.005). A similar trend was observed in mean MFR when the same analysis performed for each sex separately (0.85 vs. 1.11, *p* = 0.032 and 0.80 vs. 0.96, *p* = 0.041 for males and females, respectively). Figure 1 showed median MFR in both groups for each sex. The mean liver enzyme levels, including AST and ALT, were higher in the NAFLD group, and no significant difference in mean fasting glucose levels was observed between the two groups.

### 3.2. Severity of Fatty Liver in the NAFLD Group

Among the 53 patients, 40 had grade-1 NAFLD, seven had grade-2, and grade-3 NAFLD was observed in six individuals. There was no significant difference in MFR according to the severity of NAFLD (*p* = 0.347).

### 3.3. Prevalence of Sarcopenia According to NAFLD

When using the MFR cutoff value suggested by Kim et al., the prevalence of sarcopenia in the NAFLD group was significantly higher than in the control group (69.8% vs. 47.9%, *p* = 0.014, Table 2). In the male subgroup, the prevalence of sarcopenia in the control and NAFLD group was 77.5% and 90.9%, respectively; however, the differences was statistically non-significant (*p* = 0.124). In the female subgroup, however, the prevalence of sarcopenia in the NAFLD group and the control group differed significantly (11.8% vs. 35%, respectively; *p* = 0.041).

### 3.4. Association Between MFR and NAFLD

We investigated the association between MFR and the presence of NAFLD using logistic regression analysis. After adjusting for age, sex, BMI, and serum glucose, a negative correlation between the presence of NAFLD and MFR was observed (Table 3). The probability of the presence of NAFLD was significantly increased to an OR of 8.649 as MFR decreased after controlling for confounding variables (95% confidence intervals (CI), 1.505–49.711; *p* = 0.016).

## 4. Discussion

This study is the first to demonstrate an association between the presence of NAFLD and SMM in non-obese children and adolescents. We observed that as the MFR decreases, the risk of having NAFLD significantly increases, even after adjusting for possible confounding factors. This finding indicates that reduced SMM is an independent risk factor of pediatric NAFLD.

The association between the SMM and NAFLD observed here is consistent with previous studies. Several cross-sectional studies found that sarcopenia is closely linked to the presence and severity of NAFLD [5,10,11,12,13,20], and a longitudinal study showed that increasing SMM overtime was beneficial for either preventing or resolving NAFLD [16]. However, so far, studies have been limited to adults, most of whom were obese, because sarcopenia was originally described in elderly individuals [9], and a clear association has been reported to exist between sarcopenia and obesity [21]. However, the present study was designed to include non-obese children and adolescents.

This study has several features to discuss. First, we did not consider liver enzymes as a confounding factor, although liver enzymes were also significantly higher in the NAFLD group than in the control group (Table 1); these are more likely to be a result of NAFLD than the cause. Second, the previous Korean study demonstrated that sarcopenia was significantly related to the severity of sonography-graded NAFLD [10]; however, this could not be confirmed by our study. This may primarily be explained by bias from the small number of subjects included. Unlike previous studies on larger numbers of subjects, only seven and six sub-group participants with grade-2 and -3 NAFLD were included in this study, respectively. Lastly, when the MFR cutoff value for sarcopenia was defined as 1.155 for boys and 0.723 for girls in accordance with the Korean study [9], the prevalence of sarcopenia in the NAFLD group was significantly higher than in the control group. However, when grouped by sex, both groups showed the same tendency with regard to the prevalence of sarcopenia, but the ratio and statistical significance of sarcopenia differed (Table 2).

It could be predicted that the overall frequency of sarcopenia, especially in boys, would be high because many boys with an average age close to 10 were included as subjects (mean age; 10.8 in both control and NAFLD groups); the percentile curve pattern of the MFR reveals growth patterns that differ by gender. For boys, MFR is highest at the age of 15 and then gradually decreases or remains flat, whereas, for girls, MFR is highest at the age of 10 and then decreases through adolescence [9]. This difference is most probably rooted in hormonal differences between the sexes, since in puberty, females gain more fat mass, whereas males gain more fat-free mass [22]. Because sarcopenia has been regarded as an adults’ disease, there is a lack of age and sex-specific normative data regarding children’s SMM, and no gold standard tool to assess muscle function exists, which is an obstacle to defining childhood sarcopenia. In most adult studies, sarcopenia was defined using %ASM (%), calculated as ASM/weight (kg) × 100; however, there is no reference data regarding %ASM in children and adolescents [9]. The studies reporting on childhood sarcopenia evaluated by body composition using DEXA, BIA, computed tomography, or magnetic resonance imaging have attempted to identify indicators of sarcopenia as psoas muscle area, SMM z-score, and MFR [22]. Validated data on childhood sarcopenia are still scarce, but a study targeting Korean children and adolescents aged 10–18 years suggested an MFR cutoff value for sarcopenia, and we used that value in this study [9]. But there was a limitation to make an accurate definition of sarcopenia; the present study focused on analyzing the relationship between SMM and NAFLD by setting SMM as a continuous variable rather than defining the sarcopenia by cutoff values.

Evidence of a link between low SMM and insulin resistance has been gathered over the recent years, but the exact pathophysiology remains to be fully investigated [11,16,20,23]. The underlying mechanism is thought to be complex [5,10,11,16], and the hypotheses currently being discussed can be summarized as follows: First, considering that skeletal muscle is the major insulin-mediated glucose utilizing organ, a reduction in SMM may cause insulin resistance, which is one of the primary causes of NAFLD [10,12,24,25]. As a result of insulin resistance, a combination of increased free fatty acids and decreased lipid beta-oxidation in the liver may lead to the accumulation of triglycerides in the liver [16,24]. Second, skeletal muscle is an endocrine organ secreting various peptides referred to as myokines, such as interleukin-6 (IL-6) and irisin. In an animal model study, IL-6 showed a protective effect against NAFLD [26,27]. Irisin, an exercise-inducible myokine, plays a critical role in fatty acid beta-oxidation in the liver [11,16,28], and it has been demonstrated that serum irisin concentrations are inversely associated with the degree of fatty liver in obese humans [29]. Therefore, decreased SMM could be a risk factor for developing NAFLD by reducing the secretion of favorable myokines. Third, low SMM promotes physical disability and increases obesity, creating a vicious cycle leading to liver steatosis [16]. Fourth, The intracellular oxidative stress, one of the pathophysiology of sarcopenia, causes chronic low-grade inflammation with increased concentrations of proinflammatory cytokines such as tumor necrosis factor-alpha (TNF-α) [30,31,32] and theses cytokines may trigger to stress responses in hepatocytes, leading to TNF-α-mediated liver damage [12,30]. Fifth, low vitamin D levels are being presented as a new potential pathophysiological mechanism, as vitamin D has been reported to play a major role in maintaining muscle function, and in recent years, it has been considered a factor associated with both sarcopenia and NAFLD [33,34]. Further studies are required to support and organize these hypotheses about the relationship between low SMM and NAFLD.

A couple of study limitations should be noted. We defined NAFLD using abdominal sonography, not by liver biopsy, which is the gold standard method for diagnosing NAFLD. However, biopsies are an invasive method, and sonography is widely used to diagnose NAFLD with a sensitivity of 60%–94% and a specificity of 66%–95% [10]. We measured muscle mass using BIA, which may have limited validity when compared with DEXA. However, in recent studies, a good correlation between BIA and DEXA has been demonstrated for the evaluation of body composition, and BIA is simple, rapid, and non-invasive and, therefore, particularly applicable to children [22,35]. Due to missing data, we could not adjust for all confounding factors, potentially driving NAFLD development, including hypertension and dyslipidemia. In addition, though the study was particularly focused on non-obese subjects, the possibility of genetic factors being involved in NAFLD development could not be completely ruled out.

The present study indicates that SMM could be a significant risk factor of NAFLD in children and adolescents who are not obese. Despite the growing number of NAFLD patients, no pharmacological agents have yet been approved for the treatment of NAFLD or NASH. Thus, most NAFLD treatment strategies focus on weight loss, either by modifying lifestyle factors, such as changes in exercise and diet, or even through bariatric surgery [36]. However, in clinical cases, NAFLD is often found even in children who are not obese, and there is a limit to requiring weight loss in these cases. Based on the results of this study, it might be considered that weight training to reach the amount of muscle mass can be one of the strategies for helping prevent or improve NAFLD, rather than simply recommending exercise. Further well-designed studies are necessary to support these conclusions.

## 5. Conclusions

In this study, NAFLD was significantly associated with relatively low SMM beyond sarcopenia, in non-obese children and adolescents. The risk of having NAFLD significantly increased as MFR decreased, after adjusting with age, sex, BMI, and serum glucose. Weight training could be suggested in treatment strategies aiming to ameliorate or prevent NAFLD in non-obese children and adolescents.

## Figures and Tables

**Table 1:** Comparison of baseline characteristics between the control and NAFLD group.

Parameters	Mean ± SD or *n* (%)		
	Control (*n* = 73)	NAFLD (*n* = 53)	*p* Value
Demographics			
Age (Year)	10.8 ± 1.4	10.8 ± 1.3	0.987
Sex, Male	40 (54.8)	33 (62.3)	0.402
Anthropometrics			
Height (cm)	145.3 ± 11.2	144.6 ± 10.1	0.729
Weight (kg)	44.6 ± 7.4	45.2 ± 7.5	0.648
BMI (kg/m^2^)	21.0 ± 1.5	21.5 ± 1.3	0.057
ASM (kg)	12.0 ± 3.3	11.4 ± 3.1	0.344
MFR	1.04 ± 0.5	0.83 ± 0.3	0.005
Male	1.11 ± 0.6	0.85 ± 0.3	0.032
Female	0.96 ± 0.3	0.80 ± 0.2	0.041
Biochemistry			
AST (IU/L)	19.5 ± 3.4	33.7 ± 19.2	<0.001
ALT (IU/L)	13.3 ± 4.2	45.6 ± 42.9	<0.001
Glucose (mg/dL)	95.7 ± 6.8	98.3 ± 10.2	0.110

Data are presented as mean ± SD or number (percent). Abbreviation: NAFLD, non-alcoholic fatty liver disease; SD, standard deviation; BMI, body mass index; ASM, appendicular skeletal muscle mass; MFR, skeletal muscle-to-body fat ratio; AST, aspartate aminotransferase; ALT, alanine aminotransferase.

**Table 2:** Prevalence of sarcopenia in the control and NAFLD group.

	Control	NAFLD	OR (95% CI)	*p* Value
Sarcopenia *, *n* (%)	35/73 (47.9)	37/53 (69.8)	2.511 (1.192–5.288)	0.014
Male, *n* (%)	31/40 (77.5)	30/33 (90.9)		0.124
Female, *n* (%)	4/33 (11.8)	7/20 (35)	3.904 (0.971–15.702)	0.047

* MFR cutoff value for sarcopenia was defined as 1.155 for boys and 0.723 for girls. Abbreviation: NAFLD, Non-alcoholic fatty liver disease; OR, odds ratio; CI, confidence interval; MFR, skeletal muscle-to-body fat ratio.

**Table 3:** Logistic regression analysis for the association of NAFLD with MFR.

	Presence of NAFLD (*n* = 126)			
	B	SE	OR (95% CI)	*p* Value
MFR	2.157	0.892	8.649 (1.505–49.711)	0.016

Outcomes derived from logistic regression analysis with presence of NAFLD associated with MFR as a continuous variable, adjusting with age, gender, BMI, and serum glucose. Abbreviation: NAFLD, Non-alcoholic fatty liver disease; MFR, skeletal muscle-to-body fat ratio; B, beta; SE, standard error; OR, odds ratio; CI, confidence interval.
